# Transspecies Transmission of Gammaretroviruses and the Origin of the Gibbon Ape Leukaemia Virus (GaLV) and the Koala Retrovirus (KoRV)

**DOI:** 10.3390/v8120336

**Published:** 2016-12-20

**Authors:** Joachim Denner

**Affiliations:** Robert Koch Institute, 13353 Berlin, Germany; DennerJ@rki.de; Tel.: +49-30-18754-2800

**Keywords:** gibbon ape leukemia virus, koala retrovirus, retroviruses, transspecies transmission

## Abstract

Transspecies transmission of retroviruses is a frequent event, and the human immunodeficiency virus-1 (HIV-1) is a well-known example. The gibbon ape leukaemia virus (GaLV) and koala retrovirus (KoRV), two gammaretroviruses, are also the result of a transspecies transmission, however from a still unknown host. Related retroviruses have been found in Southeast Asian mice although the sequence similarity was limited. Viruses with a higher sequence homology were isolated from *Melomys burtoni*, the Australian and Indonesian grassland melomys. However, only the habitats of the koalas and the grassland melomys in Australia are overlapping, indicating that the melomys virus may not be the precursor of the GaLV. Viruses closely related to GaLV/KoRV were also detected in bats. Therefore, given the fact that the habitats of the gibbons in Thailand and the koalas in Australia are far away, and that bats are able to fly over long distances, the hypothesis that retroviruses of bats are the origin of GaLV and KoRV deserves consideration. Analysis of previous transspecies transmissions of retroviruses may help to evaluate the potential of transmission of related retroviruses in the future, e.g., that of porcine endogenous retroviruses (PERVs) during xenotransplantation using pig cells, tissues or organs.

## 1. Introduction

Whereas some recent transspecies transmission of retroviruses, for example, the transmission of a lentivirus from chimpanzees to humans resulting in the human immunodeficiency virus-1 (HIV-1) or the transmission of a simian immunodeficiency virus (SIV) from sooty mangabeys to humans resulting in HIV-2, have been well studied [1], other transspecies transmissions have still not been fully analysed. Most interestingly, in the case of HIV-1 and HIV-2, such transmissions happened several times, producing numerous clades of HIV-1 and HIV-2. Transspecies transmission has also been reported for gammaretroviruses, for example, the porcine endogenous retrovirus (PERV) is thought to be the result of a transmission of a murine retrovirus to pigs [2]. Since similar sequences have been found in mice, but not in rats and hamsters, it was suggested that PERVs originated from mouse endogenous retroviruses approximately three and 10 million years ago [3]. These data were later confirmed using molecular clock calibrations, revealing that the precise age of PERV is at most 7.6 × 10^6^ years old [4].

The transspecies transmission of the gibbon ape leukaemia virus (GaLV) and the koala retrovirus (KoRV) is a prime example of a transmission that is still not yet fully understood. Some captive gibbons in Thailand were found infected with GaLV, which is an exogenous gammaretrovirus that induces leukaemia in infected animals living exclusively in captivity, as wild gibbons in Thailand are not infected [5]. The koala retrovirus (KoRV) is a close relative of GaLV, and it infects koalas in Australia [6]. The fact that both viruses are closely related suggests that they may have a common origin and could be the result of a transspecies transmission of an ancestral retrovirus via one or more intermediate hosts. However, the geographical distance between Thailand and Australia, separated by the Wallace line, makes it difficult to identify the precursor virus and the original host. The Wallace line delineates Australian and Southeast Asian fauna. West of the line is the Asian ecozone, east of it is the so-called Wallacea, which includes a mixture of Asian and Australian fauna. The eastern border of Wallacea is the Lydekker line, behind which starts the Australian fauna. Many birds do not cross the Wallace line, but some animals do, such as bats and crab-eating macaques [7,8]. Searching for the precursor virus, retroviruses related to KoRV and GaLV have been described in rodents such as South East Asian mice (e.g., *Mus caroli* [9,10] and *Mus dunni* [11]), as well as in two subspecies of *Melomys burtoni* in Australia and Indonesia [12,13]. Although these viruses are close relatives, it is still difficult to explain how gibbons and koalas, living in such diverse habitats, could be infected with the precursor virus. Furthermore, only the habitat of *Melomys burtoni* in Australia and that of the koalas overlap. Since retroviruses related to KoRV/GaLV were also identified in bats [14,15], which have the ability to fly long distances, this review will analyse the hypothesis of whether these bat viruses may be the origin of KoRV/GaLV.

## 2. The Gibbon Ape Leukaemia Virus (GaLV)

GaLV is associated with hematopoietic neoplasms in captive colonies of white-handed gibbons (*Hylobates lar*). One of five known strains was isolated from gibbons suffering from granulocytic leukaemia at the Southeast Asia Treaty Organization (SEATO) Medical Research Laboratory in Bangkok, Thailand (SEATO strain), and was shown to cause chronic myelogenous leukaemia after injection into juvenile gibbons [16]. A second strain was isolated from an animal with lymphatic leukaemia at the San Francisco Medical Center (strain SF), San Francisco, USA [17,18], and a third strain was isolated from a gibbon with lymphocytic leukaemia from a colony on Hall’s Island near Bermuda (strain GaLV-H), the only GALV strain identified outside of Thailand and the USA [19,20]. A fourth strain, GaLV-Br (brain), was isolated from two healthy gibbons inoculated with brain extracts from human patients with kuru [21]. To understand the origin of GaLV, it is important to analyse whether all infected animals came from the SEATO facility in Thailand or at least had contact with GaLV-infected animals. According to recent investigations, most of the infected gibbons were directly from the SEATO facility [22]. Even in the case of the gibbons from which GALV-Br was isolated, it is thought that either the virus has its origin in animals sent from SEATO in 1963, or that the cell lines the gibbon brain was co-cultured with were contaminated with GaLV [22].

A closely related virus was isolated from a woolly monkey (*Lagothrix lagotricha*) with multiple fibrosarcomas, called first simian sarcoma-associated virus (SSAV), and then later woolly monkey virus (WMV) [23,24]. WMV is a mixture of a replication-defective transforming virus and a replication-competent helper virus [24], which has a high sequence homology and antigenic similarity with GaLV [19,20,25,26,27]. It was shown that the infected woolly monkey had close contact with an infected gibbon [22]. In addition to these strains, related GALV were isolated from a GALV-SSAV infected marmoset tumor cell line, designated GALV-MAR (Accession number U20589.1) and from an HIV-1-infected human cell line (GaLV-X) [28,29], revealing laboratory contaminations similar to the situation with the xenotropic murine leukaemia virus (MuLV)-related virus (XMRV) in human tissues [30].

When using immunological methods to investigate for GaLV infection in 79 sera from captive gibbons in 30 North American zoological institutions, the presence of antibodies against GALV antigens was revealed in 28% of the animals, indicating previous exposure to GaLV [31]. However, virus detection in gibbon blood or serum using polymerase chain reaction (PCR) or co-culture of gibbon peripheral blood mononuclear cells (PBMCs) with human cells was negative for all samples submitted and most of the animals were healthy. Hence it can be assumed that these animals were infected with GaLV, reacted with an antibody response, and succeeded in eliminating the virus. Recent investigations have demonstrated that all antibody positive gibbons were derived from the SEATO facility or had contact with infected animals [22], thus revealing that only captive gibbons, originating from the SEATO facility in Thailand, or animals having contact with them, but not free living animals, were infected and that the virus is exogenous.

## 3. The Koala Retrovirus (KoRV)

The detection and characterization of the KoRV has been well described [6,32,33]. An involvement of retroviruses in lymphoma and leukaemia in captive and free-living koalas (*Phascolarctos cinereus*) has been recognized quite early [34]. Convincing evidence was obtained when virus particles were found in mitogen-stimulated PBMCs and lymphoma tissues of hundreds of koalas and an infectious virus was then isolated and cloned [35]. Soon, it became clear that this virus may be endogenous, being integrated in the genome of normal koalas, but it may also exist as an exogenous virus [35]. Despite the taxonomical distance between gibbons and koalas, sequencing of the virus has demonstrated that KoRV is closely related to GaLV [22]. Additionally, to a lesser extent, GaLV and KoRV are also related to PERVs and MuLVs. Therefore, since a KoRV-like virus was not found in marsupials related to koalas, it seems most likely that the GaLV/KoRV grouping is the result of a relatively recent transspecies transmission of a related virus into gibbons and koalas. In this context, it is interesting to note that the first infections of koalas with the precursor virus took place in the north of Australia and the infection moved like a wave to the south of the koala habitat [36].

At present, two main subtypes of KoRV are known: KoRV-A, which uses Pit-1, a sodium-dependent phosphate transporter as a receptor; and KoRV-B and KoRV-J, both using the thiamine transport protein 1 (THTR1) (Table 1).

Since KoRV-B and KoRV-J are the result of a recombination between KoRV-A sequences with still unknown endogenous sequences, a new receptor binding domain (RBD) in the envelope protein was created and THTR1 is used instead of Pit-1. Additional, still unclassified KoRVs have also been described in previous reviews [44].

It remains unclear whether only KoRV-B or also KoRV-A are involved in tumor induction [40,42]. Like other retroviruses, including HIV-1 [45], KoRV induces severe immunodeficiencies in the infected animals, leading to serious infections, such as severe chlamydial infections [46]. Since there are effective vaccines protecting cats from infections with the feline leukemia virus (FeLV) [47], a virus related to KoRV, successful attempts have also been undertaken to generate an effective vaccine against KoRV infections [48]. Unfortunately, vaccination can be difficult in animals carrying an endogenous KoRV which produces a subsequent tolerance against the vaccine [49].

## 4. Related Rodent Viruses

The first evidence of related retroviruses was reported shortly after the description of GaLV when an endogenous retrovirus (McERV) with sequence homology to GaLV was detected in the Asian feral mouse *Mus caroli* [9,10]. Additionally, immunological assays showed that their antigens cross-reacted. Despite the similarity of the genomic sequence with GaLV, McERV has a different host range and uses a different receptor (plasmolipin), and therefore it is unlikely to be the precursor of GaLV/KoRV [50]. Endogenous retroviruses related to GaLV/KoRV were also detected in *Mus cervicolor* and in the rodent *Vandeleuria oleacea* [51,52]. This similarity was mainly evaluated using immunological and DNA hybridization techniques, however more recent full genome data showed differences between the sequences. McERV is most closely related to the *Mus dunni* endogenous virus (MDEV) [53] and the *Mus musculus* endogenous retrovirus (MmERV) [54]. Phylogenetically, these viruses form a sister clade to the GaLV/KoRV clade [55]. However, MmERV and MDEV are closer related to the GaLV/KoRV group than to the MuLV, including the Moloney MuLV, the Friend MuLV, and the Rauscher MuLV [22,54].

When 42 Australian vertebrate species were screened using PCR, in order to detect proviral sequences closely related to GaLV/KoRV, a novel gammaretrovirus was detected in an Australian rodent, the grassland melomys, *Melomys burtoni* (MbRV) [12]. MbRV shares 93% sequence homology with GaLV-SEATO and 83% identity with KoRV, a much higher homology when compared with the previously described related rodent viruses. Since the geographic ranges of the grassland melomys and koalas partially overlap, a transspecies transmission of MbRV from melomys to koalas could be theoretically possible. However, since melomys do not live in South East Asia, MbRV cannot be the origin of the GaLV, despite the fact that MbRV is closer related to GaLV than to KoRV [12].

**Figure 1:**
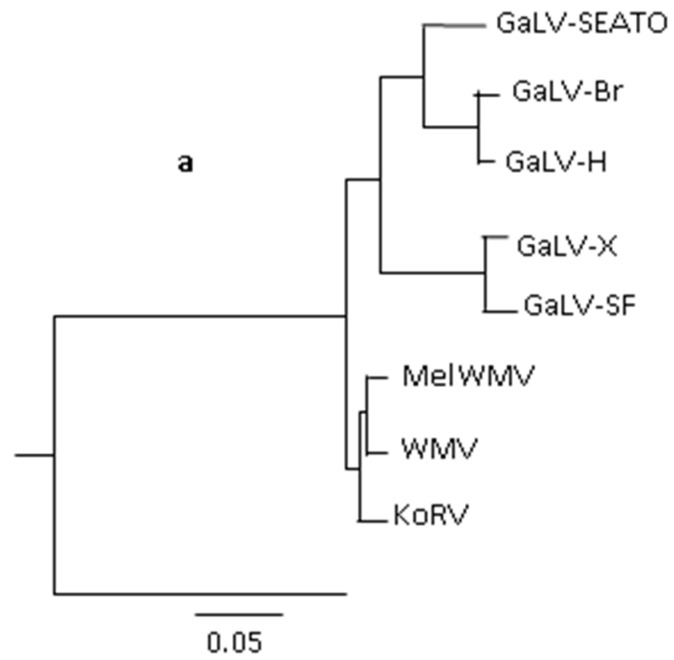

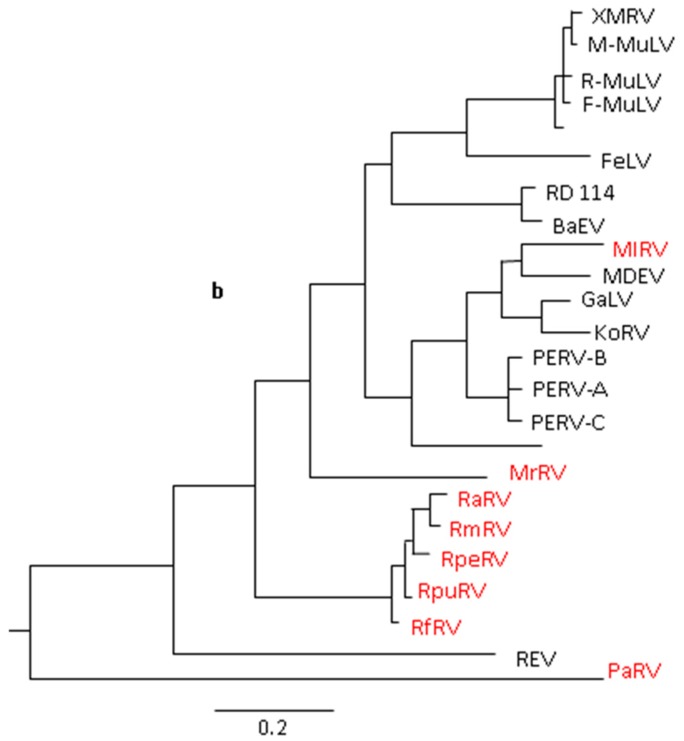
**Phylogenetic trees of gibbon ape leukaemia virus (GaLV)- and koala retrovirus (KoRV)-related viruses.** (**a**) GaLVs maximum likelihood phylogenetic tree using full genome sequences, modified after Alfano et al. [13]. GaLV-SEATO (GaLV from the Southeast Asia Treaty Organization Medical Research Laboratory in Bangkok), GaLV-Br (GaLV-Brain), GaLV-H (Hall’s Island), GaLV-X (GaLV from a human immunodeficiency virus type 1 (HIV-1) infected cell line), GaLV-SF (GaLV SanFrancisco) , *Melomys* woolly monkey virus (MelWMV) woolly monkey virus (WMV), *Melomys burtoni* retrovirus (MbRV); (**b**) maximum likelihood phylogenetic tree of the polymerase gene (amino acids) of GaLV-related gammaretroviruses modified after Cui et al. [14]. Xenotropic murine leukaemia virus (MuLV)-related virus (XMRV), prexenotropic MuLV-related virus 1 and 2; Moloney-, Rauscher- and Friend murine leukaemia virus (MuLV; M-, R-, F-MuLV); feline leukaemia virus (FeLV); feline RD114 virus (RD114); baboon endogenous retrovirus (BaEV); *Megaderma lyra* retrovirus (MIRV); *Myotis ricketti* retrovirus (MrRV); retroviruses from *Rousettus affinis*, *R. pearsoni*, *R. megaphyllus*, *R. pussillu*, *R. ferrumequinum* (RaRV, RmRV, RpeRV, RpuRV); reticuloendotheliosis virus (REV); *Pteropus alecto* virus (PaRV). Viruses marked red indicate bat gammaretroviruses.

Recently, a new gammaretrovirus, Melomys woolly monkey virus (MelWMV), was detected in another *Melomys burtoni* subspecies living in the Wallacea in Indonesia [13]. This virus was detected as a result of screening 26 Southeast Asian rodent species and all other 25 species were negative. MelWMV has 98% homology with the WMV and is a subtype of WMV, whereas MbRV from the Australian animals is a sister taxon (Figure 1a). Stop codons in *env* and *pol* indicate that it is a defective, endogenous retrovirus [13]. Since MelWMV und MbRV are the closest relatives to GaLV, they could be the origin of GaLV, although it is also possible that the two *Melomys* subspecies were infected by a GaLV-like virus from an unknown species.

## 5. Related Bat Retroviruses and Their Possible Transmission

Bats are the second largest group of mammals with approximately 1000 different species. Bats harbor more than 60 distinct emerging and re-emerging human viral pathogens, including Ebola viruses and the severe acute respiratory syndrome coronavirus (SARS-CoV) [55,56]. Interestingly, bats carry many viruses and yet they do not usually show overt signs of a disease. In addition to these exogenous viruses, numerous endogenous retroviruses have also been described in bats, however most of them have major deletions or frameshift mutations in the pol gene coding for the polymerase, indicating that they are defective. Among the endogenous retroviruses are the Megaderma lyra retrovirus (MIRV), which is from an insectivorous bat, and the Rousettus leschenaultia retrovirus (RIRV), from a frugivorous bat [14,15]. RIRV clusters with the PERVs and MIRV clusters with MDEV, KoRV and GaLV (Figure 1b). All GaLV are able to grow on CCL-88 bat lung fibroblasts [20], which serves as a strong indicator that mammalian retroviruses may have originated in bats. Additionally, numerous betaretroviruses have also been described in bats [57]. Therefore, it can be assumed that rodents and bats serve as equally important intermediate hosts for endogenous retroviruses [58,59].

## 6. Open Questions

Although the speculation that bat retroviruses represent the precursor of both the GaLV and the KoRV is highly attractive, this theory is still not proven and some important questions are still unanswered. Firstly, when was the introduction of these viruses into their host populations? GaLV was reported for the first time in the 1970s [5], and the introduction of the KoRV precursor into koalas was first dated approximately 200 years ago [35]. Meanwhile, a widespread distribution of KoRV in the late 1800s was described [60]. These findings would be consistent with a historical account that an epidemic with symptoms that may have been similar to those caused by chlamydia killed large numbers of koalas during the period 1887–1889 [61]. Secondly, why is WMV more closely related to MbRV than to GaLV, even though WMV is the result of an infection of a woolly monkey with GaLV, whereas MbRV is suggested to be the precursor of the GaLV? Thirdly, the GaLV/KoRV-like viruses found until now in the *Melomys* subspecies [12,13] and in bats [14] have been integrated into the genome of these species a long time ago and they are now defective. Based on the similarity of its 5′ and 3′ long terminal repeats (LTR), MelWMV integrated into the genome of the Indonesian melomys 200,000 years ago [13]. Therefore, it is unlikely that they are responsible for the recent infections in koalas and captive gibbons. *Melomys* can then be excluded as the origin of the GaLV because all *Melomys* species never crossed the Wallace line [13]. Modern viruses related to GaLV/KoRV from a species able to cross the Wallace line and able to infect koalas and gibbons in captivity (but not free-living gibbons) may be the best candidates for the GaLV/KoRV precursor. Fourthly, why are the gibbons in the SEATO facility infected and not animals in the wild? One explanation may be that the bats carrying the virus were attracted to food in the facility, or that infected bats were kept there in captivity.

## 7. Gammaretroviruses and Xenotransplantation

This analysis of the transspecies transmission of GaLV/KoRV-related gammaretroviruses is of great interest for the evaluation of a potential transmission of the closely related PERVs during xenotransplantation using pig cells, tissues and organs. Xenotransplantation is under development due to the increasing shortage of human cells and organs for the treatment of organ failure [62]. Whereas most of the potentially zoonotic microorganisms in pigs can be eliminated by treatment, vaccination and designated pathogen-free breeding, PERVs cannot be eliminated this way because they are integrated into the genome of all pigs. Since PERV-A, PERV-B and recombinant PERV-A/C can infect human cells in vitro, the question arises whether PERVs represent a special risk for xenotransplantation [63]. Viruses related to PERV such as GaLV [28], KoRV [39,64] and FeLV [65] can also infect human cells in vitro, however no transmission to humans in vivo has yet been reported. For example, in 204 veterinarians with a reported extensive duration of work with cats (mean, 17.3 years) and multiple high-risk exposures (e.g., cat bites, scratches, and injuries with sharp instruments), neither serologic nor molecular evidence of FeLV infection was detected [66]. Reports that another gammaretrovirus coming from mice, XMRV, infects humans and causes prostate cancer and chronic fatigue syndrome described an artifact [30]. No transmission of PERV was also observed in the first clinical xenotransplantations (more than 200 patients), in numerous pig to non-human primate transplantations, as well as in PERV infection experiments in rodents and non-human primates with and without strong pharmaceutical immunosuppression [63]. Recently, in two prospective clinical trials, transplanting pig islet cells to diabetic patients in New Zealand [67] and Argentina [68], PERV transmission was also not observed. This indicates that gammaretroviruses could potentially not be able to infect humans, despite the fact that human cells carry the corresponding receptor and can be infected in vitro.

To increase the safety of xenotransplantation, additional measures can be undertaken, such as selection of PERV-C negative pigs, in order to prevent recombination with PERV-A which would result in highly replication-competent PERV-A/C, inhibition of PERV expression by RNA interference in vitro and in vivo in transgenic pigs expressing the corresponding siRNA, and vaccines based on the envelope proteins [63]. Recently, attempts were undertaken through genome editing to inactivate all PERVs in the genome, either using a zinc finger nuclease (ZFN) [69], or clustered regularly interspaced short palindromic repeats (CRISPR) acting with CRISPR-associated nuclease 9 (Cas9) [70]. Using CRISPR/Cas, 62 proviruses were inactivated in immortalized pig cells and the question is, whether this strategy can be used to generate PERV-free animals suitable for xenotransplantation [71].

## 8. Conclusions

Transspecies transmission of retroviruses is a common event, and can be seen in various viruses, such as HIV-1, which is the result of a transspecies transmission of a lentivirus from chimpanzees to humans and in HIV-2, which is the result of a transspecies transmission of a SIV from sooty mangabeys to humans [1]. Transspecies transmission is also common for gammaretroviruses; PERV, for example, is the result of a transspecies transmission from mice to pigs [3,4]. GaLV and KoRV are also the result of a transspecies transmission of a retrovirus from a still unknown host. Related viruses have been found in rodents and bats and it is of great interest to find the precursor virus and its hosts. To study the transmission of retroviruses from one species to another is of great importance in the context of transmissions, which may occur, for example, when PERVs are transmitted to the human recipients during xenotransplantation.

## Figures and Tables

**Table 1:** Receptor usage and distribution of different koala retrovirus (KoRV) and gibbon ape leukaemia virus (GaLV).

Viruses	Receptor Usage	Distribution	Reference
KoRV-A	Pit-1	Australia	[34,35]
		Japanese Zoos	[37,38]
		German Zoos	[39]
		San Diego Zoos	[40]
KoRV-B	THTR1	Los Angeles Zoos	[40]
		Japanese Zoos	[41]
		European Zoos	[42]
		Australian wild-living koalas	[43]
GaLV	Pit-1	Gibbons; various locations, cell culture contaminations	[16,17,18,19,20,21,22,23,24,25,26,27,28,29]

THTR1: thiamine transport protein 1; Pit-1: phosphate transporter 1.
